# Estimating the Vertical Structure of Weather-Induced Mission Costs for Small UAS

**DOI:** 10.3390/s19122770

**Published:** 2019-06-20

**Authors:** John J. Bird, Scott J. Richardson, Jack W. Langelaan

**Affiliations:** 1 Department of Aerospace Engineering, Pennsylvania State University, University Park, PA 16802, USA; jjbird@psu.edu; 2 Department of Meteorology and Atmospheric Science, Pennsylvania State University, University Park, PA 16802, USA; sjr17@psu.edu

**Keywords:** UAS, atmospheric awareness, autonomy

## Abstract

The performance of small uninhabited aerial systems (UAS) is very sensitive to the atmospheric state. Improving awareness of the environment and its impact on mission performance is important to enabling greater autonomy for small UAS. A modeling system is proposed that allows a small UAS to build a model of the atmospheric state using computational resources available onboard the aircraft and relate the atmospheric state to the cost of completing a mission. In this case, mission cost refers to the energy required per distance traveled. The system can use in situ observations made by the aircraft, but can also incorporate observations from other aircraft and sensors. The modeling system is demonstrated in a flight test aboard a small UAS and validated against radiosondes and numerical weather model analyses. The test demonstrates that the modeling system can represent the atmospheric state and identifies times where significant error exists between the state expected by the numerical weather model and that observed. Transformation of the atmospheric state into a mission performance cost identifies cases where the mission performance cost predicted by a numerical weather model differs from that observed by more than 30%.

## 1. Introduction

The technology and regulatory environment surrounding small uninhabited aerial systems (UAS) is evolving rapidly, enabling more capable aircraft and longer-range and endurance missions (see for example [1] and references therein for examples specific to atmospheric sciences). Recently a UAS was designated as an air carrier in the United States, opening the door for wider commercial use [2]. However, the capable control systems developed for UAS give the impression of greater autonomy, awareness, and decision-making ability than they actually possess. Advances in sensing and understanding the environment around small UAS are needed to enable safe and reliable operations without constant direct supervision. Indeed, Barbieri et al. [3] compares meteorological sensing capabilities of 14 different types of aircraft (both rotary-wing and fixed-wing) equipped with various sensors to characterize and assess the use of small UAS for scientific measurements.

In particular, UAS need to be aware of and responsive to the atmospheric state. All aircraft are susceptible to the influence of the atmosphere, but small UAS are especially sensitive because of their slow speeds and limited power budgets. Most UAS operations today take place within line of sight of an operator who can monitor the weather conditions and aircraft state and use their expertise to make decisions for the aircraft. As UAS find use in operations requiring increased autonomy, the monitoring, and decision-making ability that has resided in the operator needs to be replicated onboard the aircraft.

A critical component of the decision-making process is situational awareness. For a small UAS, situational awareness requires integrating information from onboard sensors, other aircraft, predictive models, and remote sensing into an understanding of its surroundings. Just as traffic avoidance systems use multiple sources of information to sense the collision threat environment, efficient and safe flight planning requires sensing the atmospheric environment and assessing its effect on the aircraft’s utility and safety.

The focus of the work presented here is on development and implementation of a method to model the vertical structure of atmospheric properties directly relevant to flight performance (in this case, horizontal wind speed and direction and solar incidence) and to relate that vertical structure to the energy cost of performing the aircraft’s mission. Because the focus is on estimating the vertical profile to aid flight planning, the work presented here uses sensors that are part of a standard low-cost autopilot suite (for example, the Pixhawk module, which includes GPS, MEMS accelerometers and rate gyros, magnetometer, barometric pressure, and dynamic pressure). Please note that the use of this model in flight planning is beyond the scope of this paper.

Several authors have examined building maps of the environment around an aircraft. Lawrance and Sukkarieh used Gaussian process regression to model the winds around a small aircraft and plan optimal flight paths [4]. Depenbusch et al. used a grid of one state Kalman Filters to estimate a 2-D map of updrafts [5], permitting a small UAS to find thermals and climb without running its motor. These approaches allow accurate descriptions of complex environments and enable detailed flight planning, but scale poorly for longer-range missions.

Gaussian process regression requires maintaining and inverting a matrix with dimension equal to the number of observations, which grows unreasonably large for long flights. The grid-based approach requires a discretization as fine as the aircraft turn radius, roughly 25 m, so a map covering a large flight area would be unreasonably large for use on the aircraft. These approaches, which seek to map the lateral distribution of the environmental state, face a more fundamental problem when applied to longer-range flights. The perturbations these systems seek to model are relatively small so an observation and the decisions which it informs is invalid beyond the decorrelation scale of the atmosphere. At low altitudes, this is approximately one half of the boundary layer depth [6], of order 1 km.

Glasheen et al. evaluated high-resolution weather forecasts as a way to provide the aircraft with long-range knowledge of the environmental state [7]. Error in the position and timing of forecast phenomena makes direct comparison of high-resolution observations and forecasts challenging [8] so they compare the distributions of atmospheric properties observed by the aircraft over a mission to those forecast by a large eddy simulation. The results show systematic differences between forecast and observed conditions, demonstrating the need to use real-time observations to correct forecast error. Higher-altitude forecast error (above the tropopause) is described in [9], which discussed differences between modeled (using WRF) and measured (using a balloon-launched UAS) atmospheric conditions.

Several authors have sought to bridge this gap by assimilating observations from small UAS into a weather model [10,11]. This promises to couple observations from the aircraft into the environmental state in a dynamically consistent way, but requires constant communication between the aircraft and ground because the weather models required for data assimilation are too complicated to run onboard the aircraft. It also faces a time lag problem–the aircraft cannot make use of its observations until a subsequent model analysis or forecast is complete and transmitted back to the aircraft.

On a more fundamental level, a small number of observations may not be sufficient to correct the state of a numerical weather model, especially if an error is caused by unmodeled dynamics or large-scale error in the boundary conditions. The dimension of the atmospheric state tracked by a numerical weather model is very large, so it is not feasible to fully describe the covariance between states or between an observation and all states. While a few observations may provide meaningful information about the entire atmosphere, if the correlation between that observation and distant states is not known, then the model cannot fully reflect the impact of the observation. For instance the forecast position of a cold front has significant implications for the entire atmospheric state on a large scale, but a few UAS observations that the front is moving faster (or slower) than expected may not be enough to force adjustment in the entire field of a weather model.

Rather than trying to correct the atmospheric state, Oettershagen et al. use a potential flow model and high-resolution elevation maps to “fill in” the wind field at scales below that resolved by weather model grids [12]. This approach is promising for identifying and exploiting orographically induced winds at high resolution, but it cannot address other atmospheric properties relevant to a small UAS or incorporate observations of the true atmospheric state. It is also susceptible to errors introduced by the numerical weather forecast used to specify the boundary conditions for the flow solver.

Frew et al. describe a system for combining *a priori* forecasts with aircraft observations and remote sensing to enable intelligent response to complex atmospheric environments [13]. The approach divides the computational load between the aircraft and a ground station: the aircraft builds a Gaussian process model of its immediate environment and employs receding horizon control; the ground station fuses multiple data sources and provides long-range guidance. Only the very-near environment is modeled on the aircraft however, so for independent operations this system faces limitations similar to the systems developed by Depenbusch et al. and by Lawrance and Sukkarieh.

To enable a small UAS to operate reliably in complex atmospheric environments, its representation of the environment should exhibit several characteristics. First, the environment should be represented in a way that enables moderate- to long-range planning (several hours endurance and hundreds of kilometers range). Methods based on Gaussian process regression and the mapping methods described in [5] are currently limited to local (sub 10 km scale) flight. Second, the model should be maintained on board the aircraft and should be constructible from in situ observations to enable operation in remote locations or with unreliable communications. This limits available computational power, and methods based on data assimilation into large-scale numerical weather prediction tools are currently intractable on small single-board computers. Third, the model should be implemented in a way that permits incorporation of data from other aircraft or sensors if they are available. This will improve overall situation awareness. Fourth, since the focus here is on predicting (and ultimately improving) mission performance, the atmospheric states that strongly affect aircraft performance should be modeled, and those states should be easily transformable to a mission cost. Please note that directly modeling mission cost is less helpful because integrating information from different sensors (e.g., a stationary weather tower or an aircraft traveling in a different direction) becomes troublesome. Finally, the model should permit the use of *a priori* predictions about the state of the atmosphere, but should not require these predictions.

Key insights to enabling longer-range atmospheric awareness and distributed observation are that the atmosphere generally forms in strata and that perturbations in the atmosphere occur predominantly at very small and very large scales [14]. Small-scale perturbations (characteristic scales of 1 km or 10 min) are unresolved by numerical weather models but their small size and duration means that a cruising aircraft transits many of these perturbations and their effect on the aircraft performance averages out. Large-scale perturbations have characteristic scales of hours and hundreds of kilometers, these features are resolved by weather models but with residual error similar to that observed by Glasheen et al. [7]. Unlike the small perturbations, this error is correlated over scales of tens of kilometers so it can significantly impact the performance of a small UAS.

**Figure 1:**
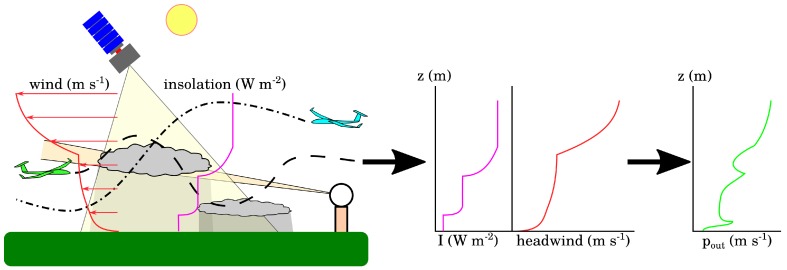
The aircraft environmental model should be able to be constructed onboard the aircraft from in situ observations but be able to incorporate observations from other platforms if available. It should model the atmospheric structure in a way that permits transformation into other quantities of interest.

This correlation permits nearby observations to be related and a one-dimensional model of the atmospheric state (i.e., variation in atmospheric state as a function of altitude) to be constructed which is valid over moderate ranges, especially if time updates can be provided by a numerical weather model. A schematic of the operation of this system is depicted in Figure 1 (a similar figure showing multiple sensor platforms for the purpose of scientific measurements in the atmospheric boundary layer is given in [15]). Each of several UAS integrates its own measurements with those of other aircraft and remote sensing systems to maintain its model of the environmental state which is then used to compute mission performance cost. While the modeling system is distinct, this communication architecture is similar to that employed by Depenbusch et al. [5] and shares the property that in the presence of perfect communications, independently constructed environmental models will be identical. Each aircraft can transform the environmental state into a measure of cost appropriate to its mission, so the aircraft uses the distributed observing system as a sensor for the vertical profile of its mission performance cost. Note the difference in focus between this work and past efforts in UAS weather observation: here the focus is on developing a model of the atmosphere that can be used in UAS mission planning; Jacob et al. [15] focuses on scientific measurements.

Because most measures of aircraft capability can be related to the energy available to complete a mission, this work will adopt the power required to travel at a specified ground speed in a desired direction as a cost function. This work will focus especially on a system designed for a small UAS equipped with a solar array to enhance its endurance, so relevant atmospheric properties are the wind vector and solar insolation. Section 2 describes the use of splines to model variation in the parameters of interest and implementation of a Kalman filter to track the time evolution of those parameters and incorporate measurements. Section 3 describes computation of the travel cost (i.e., energy required per distance traveled). Section 4 describes results of simulations that address solar insolation and atmospheric modeling using multiple aircraft. Section 5 describes the Vulture small UAS and results of flight tests conducted using this aircraft, and discusses comparison of flight test results with data from balloon sondes and High-Resolution Rapid Refresh (HRRR) forecasts. Finally, Section 6 presents concluding remarks.

## 2. Basis Splines for Environmental Modeling

There are several techniques that can be used to reconstruct an unknown function from noisy samples. Gaussian processes are a common general-purpose approach, but scale poorly with large numbers of measurements, do not handle degradation of information well, and are difficult to update in real time on single-board computers that can be carried on a small UAS. Their basic concept is powerful however, estimating the influence of several basis functions on the overall value of a function. Specifying these basis functions *a priori* offers one way to resolve the issues with Gaussian process regression in this application while still offering the ability to represent complex functions whose shape is not known.

Polynomial spline functions are commonly used to interpolate unstructured data as they can represent arbitrary functions [16]. A spline is specified by a series of “knots,” or control point locations, and the order of derivative which is permitted to have discontinuities at the knots. Between knots the spline follows a polynomial of the chosen order. For instance, a first-order spline is piecewise linear between knots with discontinuous derivatives at the knots. Cubic splines are commonly used and offer $C^{2}$ continuity at the knots.

The exact mathematical representation of a spline can be expressed in several ways, including as piecewise continuous polynomial functions. For the purposes of recursively building an environmental model, basis splines are the most convenient. In basis spline, or B-spline form, a series of spline functions are defined covering the domain of the model. Each spline has limited support near a particular knot and the spline basis functions sum to one at each point in the domain of the model, which is [16]:

(1) $$\sum_{i=0}^{n+k-1}N_{i}\left(z\right)=1\;\forall \;z\in \left[z_{knot,min},z_{knot,max}\right]$$

where $N_{i}\left(z\right)$ is the value of basis *i* at point *z*, *n* is the number of knots in the spline and *k* is the spline order. At a desired point *z*, the value of a function can be represented as a linear combination of the spline functions. Coefficients, $c_{i}$ specify the influence of each spline basis:

(2) $$f\left(z\right)=\sum_{i=0}^{n+k-1}N_{i}\left(z\right)c_{i}=N\left(z\right)c$$

where N(z) is a row vector of the spline basis functions evaluated at point *z* and c is a column vector of spline coefficients. A spline can thus be thought of as a mapping from an n+k−1 vector to a scalar value *f* within some range $z\in [z_{knot,min},z_{knot,max}]$. Finding a model to fit a function requires finding the spline coefficient vector which minimizes the difference between the spline model and function values.

With three atmospheric parameters to model, three sets of spline coefficients will have to be estimated, representing the wind vector components and fraction of the clear-sky solar insolation which is available. The fraction of the clear-sky insolation will be referred to as solar fraction, α, it is chosen over the actual insolation to remove the effect of celestial dynamics from the model. To simplify calculations, each spline will use the same order and basis, although different bases could be used if required to provide additional model resolution for each parameter in different areas.

Equation (2) shows that the value of a spline model evaluated at a point is determined by a linear combination of the bases. If a process to be modeled is assumed to be represented by spline coefficients c, then an approximate model can be constructed from noisy observations $y_{j}=N\left(x_{j}\right)c+\nu$ where ν is noise in the observation [17]. This is a classic linear model fitting problem; it could be solved for a batch of observations using least-square estimation:

(3) $$\hat{c}={\left(N^{T}\left(z\right)N\left(x\right)\right)}^{-1}N^{T}\left(z\right)y\approx c$$

**Figure 2:**
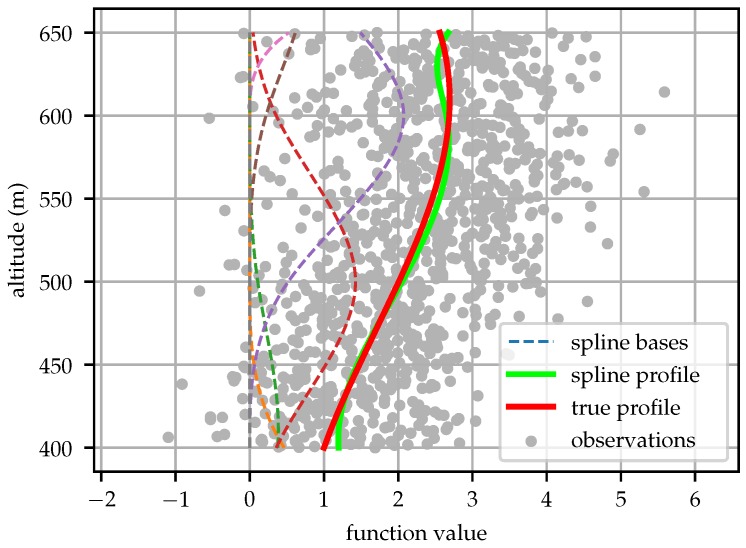
Basis splines can be used to approximate functions by a linear combination of the bases at each point. The coefficients for this example model were determined by a least-square fit of the data sampled from the equation $f\left(z\right)=-3{\left(\frac{z}{250}-400\right)}^{3}+2{\left(\frac{z}{250}-400\right)}^{2}+\exp\left(\frac{z}{250}-400\right)+\sin\left(\frac{z}{250}-400\right)$ with noise ν∼N(0,1.0). Spline bases are shown scaled by their respective coefficients.

Figure 2 shows an example of spline bases and a function approximated by a linear combination of the bases with the coefficients determined by least-square fit of noisy data. The example function fit in this instance was $f\left(z\right)=-3{\left(\frac{z}{250}-400\right)}^{3}+2{\left(\frac{z}{250}-400\right)}^{2}+\exp\left(\frac{z}{250}-400\right)+\sin\left(\frac{z}{250}-400\right)$, chosen arbitrarily to include components from several transcendental functions. One thousand samples were drawn with Gaussian noise ν∼N(0,1.0).

For an aircraft, the environment is dynamic and the coefficients c can be viewed as states that change in time. Since the basis spline is linear, the coefficient vector can be estimated recursively using a Kalman filter as observations of the environment are received [17]. A simple version of this model was used to determine the wind shear value for a dynamic soaring aircraft [18].

### 2.1. Time Update

Predictions of the evolution of the atmospheric state can be used to update the spline model. Since several splines influence the value of the model at each point, updating the spline coefficients is not as simple as applying the rate of change of the atmospheric state at a knot location to the corresponding coefficient. Writing the state of interest generically as *f*, the state derivative is related to the coefficient derivatives:

(4) $$\frac{\partial f(z)}{\partial t}=\frac{\partial N(z)c}{\partial t}=\frac{\partial N(z)}{\partial t}c+N\left(z\right)\frac{\partial c}{\partial t}$$

If the spline knot locations are to remain constant, then $\frac{\partial N(z)}{\partial t}=0$. This may not be the case if the knot locations are permitted to change, which may be used to provide greater knot density at the boundary layer top or some other region of interest which is not fixed in space. For this work the knot locations are left constant.

If the atmospheric state derivatives are available at discrete points (e.g., at the grid locations of a numerical weather prediction model) then the best-fit derivative of the coefficient vector can be computed using the pseudoinverse:

(5) $$\frac{\partial c}{\partial t}={\left(N^{T}\left(z\right)N\left(z\right)\right)}^{-1}N^{T}\left(z\right)\frac{\partial f(z)}{\partial t}$$

Please note that for the pseudoinverse to be nonsingular the derivative must be known at n+k−1 points, and also there must be a sample point $z_{n}$ in the support of every spline basis.

If time derivatives of the atmospheric state are not available, it can be modeled simply as a random walk. In this work, the HRRR numerical weather prediction model is used to determine the time derivative of the expected environmental state [19]. Time derivatives are determined by a central finite difference of the HRRR state at the observation time with a step size equal to half the HRRR output time interval.

### 2.2. Process Noise

Since the atmosphere is an uncertain environment it is desirable to have an estimate for both the mean and variance in the environmental state (and thus mission performance) cost profile, making it important to determine an appropriate value for the process noise. The model framework chosen and information available will influence the meaning and choice of process noise. If a prediction of the evolution of the atmospheric state is available the process noise represents the growth of error in the forecast with time. If a prediction of the state evolution is not available then the process noise should be chosen to represent the rate of change of the state in a random walk.

**Figure 3:**
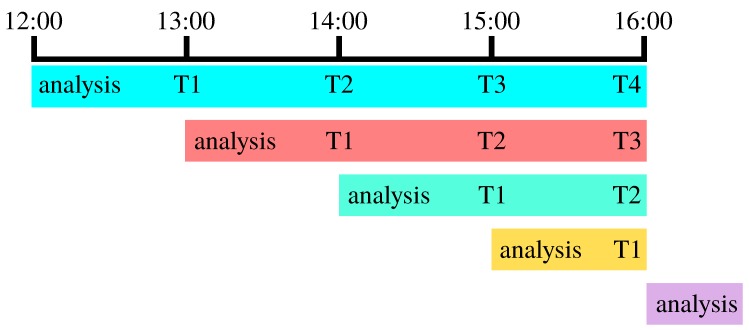
The forecast error at each hour is computed using a triangular scheme and the analysis from subsequent forecast times as “truth.” For instance, the one-hour forecast error for the 12:00 UTC forecast is the value of the desired fields from the 12:00 UTC T1 step minus the analysis value from the 13:00 UTC forecast.

To determine an appropriate process noise, the HRRR analysis and forecast was obtained for 900 forecast periods. In engineering parlance, the analysis step of a weather model is the “measurement update,” observations from weather sensors are used to update the model and provide a best estimate of the current state of the atmosphere before beginning a forecast. Figure 3 illustrates how the analysis step of subsequent forecasts is used as “truth” to compute forecast error, the difference between subsequent forecasts valid at the same time. For instance, given an hourly forecast initialized at 12:00 UTC, the one-hour forecast error is the difference between the forecast for T0+1 h and the analysis from a run initialized at 13:00 UTC, the two-hour error is the difference between the forecast for T0+2 h and the analysis from the 14:00 UTC run, and so on.

**Figure 4:**
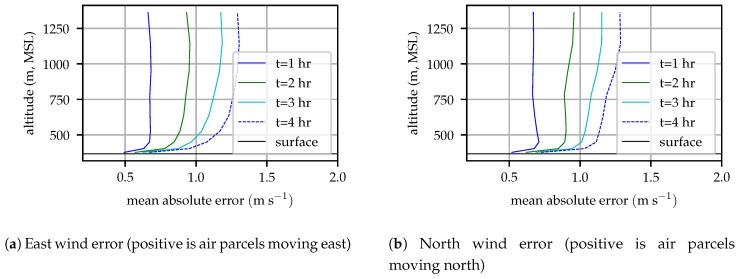
Mean absolute error profiles for the HRRR wind forecast at a point in central Pennsylvania.

The mean forecast error can then be computed by averaging the one-hour, two-hour, etc. error over many forecast periods. Figure 4 illustrates the mean wind error versus forecast hour for several model levels at an example point in central Pennsylvania.

Figure 4 clearly shows that the forecast error varies with height, with the greatest variation in the lowest 100 m. Since most aircraft cruise at altitudes above 100 m, this work uses a constant value of 0.95 $m^{2}\;s^{-2}\;h^{-1}$ for process noise, the wind magnitude variance computed from the mean 1 h forecast error.

Solar insolation is only available in the model output at the surface. The mean squared error in the surface solar fraction 1 h forecast, 0.09, is computed using the same method and used to determine the process noise at all levels.

For very large flight areas that cross regions with significantly varying atmospheric dynamics, a process noise map could be constructed which allows the process noise to vary with position and perhaps with time of day and year. Again, to simplify the filter in this work, a single static value is used.

If environmental process noise is constant with altitude then it can easily be related to the spline coefficients process noise. Since the spline functions sum to 1 at all points in the domain, a uniform increase in uncertainty can be accomplished with a diagonal process noise matrix.

(6) $$Q=I_{n+k-1}Q$$

where $I_{n+k-1}$ is the identity matrix with dimension n+k−1 and *Q* is the variance of the process noise.

If the process noise is permitted to vary with height then the forecast error growth must be transformed into error growth in the spline coefficients. Since the spline model is a linear transformation applied to the spline coefficient vector, if the covariance of the coefficient vector is known, then the variance of any point in the spline model can be computed:

(7) $$P_{f}\left(z\right)=N\left(z\right)\Sigma_{c_{f}}N^{T}\left(z\right)$$

Given the growth rate of the variance in the modeled, $\frac{\partial P_{f}\left(z\right)}{\partial t}$, the time derivative of the spline coefficient covariance can be computed in a manner analogous to the computation of the coefficient derivatives from Equation (5):

(8) $$\frac{\partial P_{f}\left(z\right)}{\partial t}=\frac{\partial N(z)}{\partial t}\Sigma_{c_{f}}N^{T}\left(z\right)+N\left(z\right)\frac{\partial \Sigma_{c_{f}}}{\partial t}N^{T}\left(z\right)+N\left(z\right)\Sigma_{c_{f}}\frac{\partial N^{T}\left(z\right)}{\partial t}$$

Assuming that the spline bases themselves are stationary, then the second term is the only one which is not trivially zero. Applying the pseudoinverse to the basis functions in Equation (8), the time derivative of the spline coefficients can be computed:

(9) $$\frac{\partial \Sigma_{c_{f}}}{\partial t}={\left(N^{T}\left(z\right)N\left(z\right)\right)}^{-1}N^{T}\left(z\right)\frac{\partial P_{f}\left(z\right)}{\partial t}N\left(z\right){\left(N^{T}\left(z\right)N\left(z\right)\right)}^{-1}$$

As in Equation (5), the derivative, $\frac{\partial P_{f}\left(z\right)}{\partial t}$ must be known at least n+k−1 points with some points in the support of all spline bases. Given a Kalman filter integration step size $\Delta t_{kf}$, the process noise can be approximated:

(10) $$Q\approx \Delta t_{kf}\frac{\partial \Sigma_{c_{f}}}{\partial t}$$

### 2.3. Observation Models

The three atmospheric states that are modeled are the north and east components of wind and solar insolation. Recall that solar insolation is modeled using solar fraction α, the fraction of expected solar insolation. The expected solar insolation can be calculated on board using a celestial model and the time of day, time of year, and the aircraft’s latitude and longitude. Throughout this work the Python PVLib library is used to compute the nominal solar insolation from celestial mechanics [20]. Please note that the expected solar insolation could also be provided by a numerical weather prediction tool that provides expected cloud cover.

These three measured quantities thus provide the observation model:

(11) $$w_{north}\left(z\right)=N\left(z\right)c_{wn}+noise$$

(12) $$w_{east}\left(z\right)=N\left(z\right)c_{we}+noise$$

(13) $$\alpha \left(z\right)=N\left(z\right)c_{\alpha }+noise$$

where $c_{\left(\cdot \right)}$ defines the weights for the basis functions for each quantity of interest. Recall that the basis functions N do not have to be the same for each quantity, although in this work they are the same. Noise is discussed in the next section.

The basis functions evaluated at the point of interest form the observation model. They represent the influence that each coefficient has on the model value at the observation point.

Sometimes multiple observations might be available simultaneously. This could happen when receiving a wind profiler measurement, a batch of observations from nearby aircraft, or a model analysis. This observation model can be extended to incorporate multiple simultaneous observations by writing an extended basis matrix as the observation model. Using the notation of Equation (2):

(14) $$f=N\left(z\right)c_{f}+noise$$

where N is an m×(n+k−1) matrix with *m* the number of observations available.

### 2.4. Measurement Noise

Measurement error should include two components–noise introduced by the equipment used to make observations and noise introduced because the point sampled is not representative of the environment as a whole. To clarify the distinction the first will be referred to as sensor noise and the second as sample noise. In this work the aircraft is estimating the wind speed and solar fraction, so the measurement noise for these properties is developed.

Sensor noise is relatively straightforward to define. The sensors themselves can be characterized to determine their measurement variance. In some cases, a measurement may be synthesized from several sensors (e.g., solving the wind triangle to compute the wind vector requires measuring the inertial and air relative velocity vectors). In this case, each sensor individually can be characterized, and the overall error be determined by propagation of error.

Sensor noise in the wind vector has been studied by several authors and techniques to compute it are well established [21,22].

Because the solar intensity is inferred from the power produced by the solar array, cloud fraction measurements are influenced by the solar array power electronics, current and voltage monitoring equipment, and aircraft attitude. Here we assume that voltage and current can be measured very accurately so that sensor error in the solar fraction is dominated by error in the aircraft’s attitude. Approximating the solar array as a flat plate in the plane of the aircraft x−y axis, the sensitivity of solar fraction to attitude error can be determined:

(15) $$\frac{\partial \alpha }{\partial \Theta }=\frac{\partial }{\partial \Theta }\frac{I_{observed}\cos\hat{i}}{I_{clear\;sky}\cos i}=\frac{I_{observed}}{I_{clear\;sky}\cos i}\frac{\partial \cos\hat{i}}{\partial \Theta }$$

where *i* is the true angle between the aircraft −z axis and the sun, and $\hat{i}$ is the expected angle based on the sensed aircraft attitude, $I_{clear\;sky}$ is the clear-sky solar insolation, and $I_{observed}$ is the observed solar insolation. Please note that *I* here is distinct from the identity matrix, I from Equation (6). Noting that cosi is the dot product of the aircraft −z axis resolved in inertial axes with a unit vector to the sun, the aircraft −z axis can be transformed by the body-to-inertial coordinate rotation [23] and cosi can be written:

(16) $$\cos i=\left[\begin{array}{ccc} s_{x} & s_{y} & s_{z} \end{array}\right]\left[\begin{array}{c} \sin\phi \sin\psi +\cos\phi \sin\theta \cos\psi \\ -\sin\phi \cos\psi +\cos\phi \sin\theta \sin\psi \\ \cos\phi \cos\theta \end{array}\right]$$

where $s_{x}$, $s_{y}$, and $s_{z}$ are the components of the unit vector to the sun. Abbreviating sin· as s· and cos· as c·, the derivative of Equation (16) with respect to the aircraft attitude is:

(17) $$\left[\begin{array}{ccc} \frac{\partial }{\partial \phi } & \frac{\partial }{\partial \theta } & \frac{\partial }{\partial \psi } \end{array}\right]\cos i=\left[\begin{array}{ccc} s_{x} & s_{y} & s_{z} \end{array}\right]\left[\begin{array}{ccc} c\phi s\psi -s\phi s\theta c\psi & c\phi c\theta c\psi & s\phi c\psi -c\phi s\theta s\psi \\ -c\phi c\psi -s\phi s\theta s\psi & c\phi c\theta s\psi & s\phi s\psi -c\phi s\theta c\phi \\ -s\psi c\theta & -c\phi s\theta & 0 \end{array}\right]$$

The measurement variance can be approximated $\sigma_{\alpha }^{2}\approx \frac{\partial \alpha }{\partial \Theta }\Sigma_{\Theta }$. Equations (15) and (17) give $\frac{\partial \alpha }{\partial \Theta }$. Assuming that attitude errors are uncorrelated and that $\cos\hat{i}\approx \cos i$, the measurement variance can be written:

(18) $$\sigma_{\alpha }^{2}=\frac{I_{observed}}{I_{clear\;sky}\cos\hat{i}}\left[\begin{array}{ccc} \frac{\partial }{\partial \phi }\cos\hat{i} & \frac{\partial }{\partial \theta }\cos\hat{i} & \frac{\partial }{\partial \psi }\cos\hat{i} \end{array}\right]\left[\begin{array}{c} \sigma_{\phi }^{2}\\ \sigma_{\theta }^{2}\\ \sigma_{\psi }^{2} \end{array}\right]$$

### 2.5. Sample Noise

The other component of noise in an observation is the sample noise. This is variance introduced because even an observation taken by a perfect sensor may not be perfectly representative of the underlying mean atmospheric state. For instance, the wind vector is influenced by convective motion, terrain interactions, and waves propagating through the atmosphere [24]. Many of these phenomena are short-lived and localized so that they appear as noise to a cruising aircraft. The effect of sample variance for solar fraction is even starker–when flying under scattered clouds the solar insolation “blinks” between sunny and cloudy, never taking its mean value.

#### 2.5.1. Wind Sampling Error

The wind sampling error can be analyzed by separating the wind into a mean and turbulent component [24,25]:

(19) $$u=\bar{u}+u^{\prime }$$

where, by definition, the mean of the turbulent component, $\bar{u^{\prime }}$, is 0 [24]. The degree to which any realization of the wind differs from the mean wind will be described by the statistics of the turbulent component. The variance of the turbulent wind motion gives the desired contribution to the measurement noise.

Scaling laws can be used to relate turbulent wind variance at different altitudes and budget equations can be used to predict the time evolution of the variance [24]. These approaches are difficult to use in flight however; scaling laws require *a priori* knowledge of the turbulent wind variance at some point and solving budget equations requires running a numerical weather model. From a practical perspective it is simpler to provide the aircraft with a weather forecast which includes a prediction of the turbulent wind variance. One commonly forecast parameter than can be used to approximate the turbulent wind variance is the mean turbulent kinetic energy (TKE) per unit mass of air, defined [24]:

(20) $$\frac{TKE}{m_{air}}=tke=\frac{1}{2}\left(\bar{{\left(u^{\prime }\right)}^{2}}+\bar{{\left(v^{\prime }\right)}^{2}}+\bar{{\left(w^{\prime }\right)}^{2}}\right)$$

where $\bar{{\left(u^{\prime }\right)}^{2}}$, $\bar{{\left(v^{\prime }\right)}^{2}}$, and $\bar{{\left(w^{\prime }\right)}^{2}}$ are the variance of the turbulent east, north, and up wind components respectively. The *u* and *v* components are assumed to have the same statistics [24]. Depending on where the mean and turbulent scale division is drawn, the variance of $u^{\prime }$ and $v^{\prime }$ can be significantly larger than $w^{\prime }$ [26,27]. When tke is drawn from a weather model it represents the variation in motion that occurs at a scale below the resolved scale of the model. Large eddy simulations and turbulence theory indicates that these subgrid-scale turbulent motions are approximately isotropic [28,29] so the sampling error for wind components can be approximated:

(21) $$R_{wind,\;sampling}\approx tke\frac{2}{3}$$

If no derivative information is available to propagate the atmospheric state forward in time, then $R_{wind,\;sampling}$ should be specified to include the effect of small mesoscale perturbations in the sampling error. This additional sampling error could be approximated by computing the variance of the wind forecast by a numerical weather prediction model over a representative mission, similar to the analysis done by Glasheen et al. [7] In this work, the wind sampling error will be determined solely by tke.

Since this work is intended to permit correction of error in *a priori* predictions of the atmospheric state, the use of a weather model to provide estimates of tke seems slightly self-defeating. The advantage offered by this system is that incorrectly specified sampling error has less effect on the aircraft’s knowledge of environment than error in the forecast atmospheric state. Incorrect specification of the sampling error could lead the Kalman filter to converge too slowly (if the sample error is too large) or provide a state estimate which is noisy (if the sample error is too small) but the state estimate will be unbiased. In contrast, reliance on an *a priori* specified model will almost certainly introduce systematic error into the aircraft’s understanding of the environment which cannot be corrected in flight.

Please note that methods to measure turbulence in flight have been described in [30,31]. Accurate measurement of turbulence requires a multi-hole probe [31], and it is in principle possible to use in situ measurements of turbulence for wind sampling error. Assessing potential improvement in performance by incorporating in situ measurement of turbulence over the use of modeled tke is beyond the scope of this paper.

#### 2.5.2. Solar Sampling Error

Assuming that clouds are scattered randomly so that a given sample point has a cloud above it with probability $f_{c}$, then cloud coverage is defined by a Bernoulli distribution with parameter $f_{c}$. The mean cloud coverage is exactly defined by the cloud fraction, and the variance in cloud cover can be determined from the variance of the Bernoulli distribution [32].

(22) $$\mathrm{Var}\left(f\right)=f_{c}\left(1-f_{c}\right)$$

If all clouds block the same fraction of incident solar energy, α, then sampling error for the solar fraction is easily defined as a simple scaling of the cloud fraction variance:

(23) $$R_{solar,\;sampling}=\alpha f_{c}\left(1-f_{c}\right)$$

Bernoulli distributed observation noise violates the assumption of Gaussian measurement noise inherent to the Kalman filter, but in numerical experiments the estimated value for α is quite close to the true value. In reality, clouds are not hard-edged and can be multi-layered so that the observation noise is not truly Bernoulli distributed. The effect of these effects should make the true observation noise closer to a Gaussian distribution by the central limit theorem. As with tke, the cloud fraction can be determined from a weather forecast provided to the aircraft *a priori* and updated in flight if enough bandwidth is available.

### 2.6. Summary of Kalman Filter Equations

The Kalman filter is implemented in discrete time using system equations

(24) $$c_{l}=Ac_{l-1}+B\frac{\partial f(z)}{\partial t}+N\left(0,Q\right)$$

(25) $$f_{l}=N\left(z\right)c_{l}+N\left(0,R\right)$$

Here *l* denotes the time index and N(0,X) denotes a Gaussian random variable with mean zero and covariance *X*. The subscript denoting the specific variable of interest has been omitted for clarity. A is the discrete time state update matrix: if the knot locations do not change with time then $A=I_{n+k-1}$ (i.e., identity). The input matrix $B=\Delta t_{kf}{\left(N^{T}\left(z\right)N\left(z\right)\right)}^{-1}N^{T}\left(z\right)$ (Equation (5), converted to discrete time using forward Euler integration). If atmospheric state derivatives are not available then $\frac{\partial f(z)}{\partial t}=0$. Process noise Q is defined in Equation (6) (if process noise is constant with altitude) and Equation (10) (if process noise varies with altitude). Measurement noise variance $R=R_{sensor}+R_{sampling}$, and the measurement covariance matrix R is diagonal for uncorrelated measurements. Please note that sensor noise for measurements taken from different platforms will be uncorrelated, but sampling noise might not be uncorrelated.

Please note that measurements from multiple vehicles are incorporated as measurements at multiple altitudes. Depending on the implementation chosen for the Kalman filter, the size of the matrix inverse scales with the number of measurements obtained at each time step. For a single aircraft this is a simple scalar inverse, and for multiple aircraft one can now choose to either design the computational hardware to fit available measurements or one can choose several aircraft/measurement sources to fit the available computational resources.

## 3. Computing Cost Profile

For an aircraft, operator, or supervising system to make intelligent decisions in response to the observed environment, the atmospheric state must be related to some measure of mission performance cost. If the entire atmospheric model can be easily converted into a model for cost, it can support efficient evaluation of flight plans since the atmospheric profile need not be sampled and transformed repeatedly. Because the atmospheric state is known imperfectly, it is desirable to determine both the expected cost and its variance.

As discussed in Section 1 the cost function adopted in this work is the power which must be used from an onboard store of energy to travel in a specified direction at a specified inertial speed. We will write all power terms as aircraft-weight specific power (i.e., $p=\frac{P}{mg}$). This gives kinematic meaning to the cost, for example the specific excess aerodynamic power represents the aircraft’s vertical rate [33].

For linear transformations, a transformation matrix can relate the spline model for atmospheric state to a spline model for mission cost. The effect of solar fraction on the aircraft’s energy budget can be determined through a scalar linear transform:

(26) $$\begin{array}{cc} j_{solar} & =T_{j/solar}c_{solar}=-c_{solar}S_{pv}\eta_{pv}I_{clear\;sky}\cos i\\ & \Rightarrow T_{j/solar}=-S_{pv}\eta_{pv}I_{clear\;sky}\cos i \end{array}$$

where $c_{solar}$ are the coordinates of the solar fraction spline and $T_{j/solar}$ is the transformation matrix from solar fraction to cost, $S_{pv}$ is the solar array area, and $\eta_{pv}$ is the solar array efficiency. The cost is negative indicating that solar energy input is beneficial.

Nonlinear transformations from the environment to cost are more complicated to perform but can be accommodated. The effect of wind on power required to achieve an inertial velocity requires this treatment. First, the required airspeed magnitude is computed from the wind and desired travel direction and speed:

(27) $$v\left(z\right)=\sqrt{\left({\bar{v}}_{n}^{2}+{\bar{v}}_{e}^{2}\right)+c_{n}^{T}N^{T}\left(z\right)N\left(z\right)c_{n}+c_{e}^{T}N^{T}\left(z\right)N\left(z\right)c_{e}-2N\left(z\right)\left(c_{n}{\bar{v}}_{n}+c_{e}{\bar{v}}_{e}\right)}$$

where ${\bar{v}}_{e}$ and ${\bar{v}}_{n}$ are the average ground speeds required to meet an outer loop goal resolved into east and north components. The required airspeed must be related to the specific power required through the aircraft’s aerodynamic performance:

(28) $$p_{output}\left(z\right)=\frac{w_{s}\left(v\left(z\right)\right)}{\eta_{propeller}}$$

where $w_{s}\left(v\right)$ is aircraft’s speed polar, relating specific power required to airspeed. For a fixed-wing aircraft this is the sink rate in an unpowered glide as a function of airspeed [34].

To determine the spline coefficients of the component of cost due to wind, Equation (28) can be computed at some altitudes $z_{l}\in \left\{z_{0},z_{1},\ldots ,z_{L}\right\}$ where L>(n+k−1) so that:

(29) $$N\left(z\right)j_{wind}\approx \frac{w_{s}\left(v\left(z,c_{n\;wind},c_{e\;wind}\right)\right)\sqrt{\sigma }}{\eta_{propeller}}$$

Which can be solved in the least-square sense to compute wind cost coefficients, $j_{wind}$:

(30) $$j_{wind}={\left(N^{T}\left(z\right)N\left(z\right)\right)}^{-1}N^{T}\left(z\right)\frac{w_{s}\left(v\left(z,c_{n\;wind},c_{e\;wind}\right)\right)\sqrt{\sigma }}{\eta_{propeller}}$$

Computation of the covariance of the wind cost coefficients is a little more challenging. In principle, the Jacobian of Equation (30) can be found and used to compute a linearized transformation of the covariance. The derivatives are laborious to compute, however. If the aerodynamic model, $w_{s}\left(v\right)$, is an empirical model then there may not be analytic derivatives at all. For this reason, it is easier to transform the covariance using the unscented transform [35].

Assuming that all costs are expressed as splines using the same knot locations, then the total cost can be determined by simply adding the coordinate vectors together:

(31) $$j=j_{1}+j_{2}+\ldots +j_{M}$$

where $j_{i}$ are the costs due to various components of the atmospheric state. For linear transformations from the environmental state to cost, the transformation matrices can be used to compute the cost variance.

(32) $$P_{j,i}=T_{j/i}P_{i}T_{j/i}^{T}$$

where $P_{i}$ is the covariance matrix of the model for state *i* and $T_{j/i}$ is the transformation from the state *i* to the mission performance cost. For the cost model considered here the total cost covariance can be written:

(33) $$j=j_{solar}+j_{wind}$$

The covariance of the cost due to wind computed using a nonlinear covariance propagation technique can then be added to the covariance due to solar fraction to determine a total covariance for the output power model.

(34) $$P_{j}=T_{j/solar}P_{j,solar}T_{j/solar}+P_{j,wind}$$

Equations (33) and (34) together provides a model for the mean and uncertainty in the aircraft mission performance cost. The environmental model and environment-to-cost transformations provide a system which allows distributed sensing of the vertical structure of the cost for an aircraft to perform a mission.

## 4. Simulation Test

Simulation results are presented to address: (1) performance of the solar insolation model, which cannot be tested in flight under FAA Part 107 (it would require flight in and above clouds); (2) the utility of incorporating data from multiple aircraft (note that the radiosonde data in the next section is used for validation). The environmental modeling system was implemented in Python using the Numpy and SciPy libraries. A longitudinal simulation of a small fixed-wing UAS was also implemented and used to test the environmental modeling system. Because the aircraft dynamics have characteristic timescales of order seconds while the atmosphere has a much longer timescale, the aircraft dynamics are neglected. The aircraft is thus represented as a kinematic particle with states: north position, east position, and altitude. It is assumed that the aircraft controller maneuvers the aircraft to a constant ground track, $\psi_{track}$ at a groundspeed of 15 $\;m\;s^{-1}$. Inputs are speed command, power output from the battery, north and east winds, and solar power input. The state equations are given:

(35) $$\frac{\partial }{\partial t}\left[\begin{array}{c} x_{north}\\ x_{east}\\ h \end{array}\right]=\left[\begin{array}{c} v_{north}\\ v_{east}\\ \dot{h} \end{array}\right]=\left[\begin{array}{c} v_{ias}\cos\psi +wind_{north}\\ v_{ias}\sin\psi +wind_{east}\\ w_{s}\left(v_{ias}\right)+\left(\tilde{p}+\frac{S_{pv}\eta_{pv}I\cos i}{mg}\right)\eta_{propeller} \end{array}\right]$$

The airspeed and aircraft heading commands, $v_{ias}$ and ψ respectively, are chosen to maintain the ground track $\psi_{track}$ at an inertial speed of 15 $\;m\;s^{-1}$. The commanded battery power, $\tilde{p}$ is generated by a proportional-integral control loop which tracks a desired altitude with a saturation climb or descent rate of $0.5\;\;m\;s^{-1}$. The system dynamics given by Equation (35) are integrated using a fourth-order Runge–Kutta solver with a 0.1 s timestep.

The atmospheric state is interpolated from the HRRR weather model to determine the wind vector and solar insolation. The standard HRRR output does not provide solar insolation at every model level, but does provide cloud fraction. The solar insolation is determined assuming that the cloud coverage at every level is independent and that all clouds block the same amount of incident sunlight so that the fraction of solar energy available is

(36) $$\alpha \left(z\right)=1.0-\left(1.0-\alpha_{surface}\right)\frac{cloud(z)}{cloud_{surface}}$$

where $\alpha_{surface}$ is the fraction of clear-sky solar insolation which is received at the surface, cloud(z) is the fraction of sky covered in cloud above altitude *z*, and $cloud_{surface}$ is the fraction of the sky which is covered in cloud at any altitude.

A mission is simulated beginning at Eastern West Virginia Regional Airport (39.3977${}^{\circ }$ N, 77.9978${}^{\circ }$ W) and traveling 50 km in the direction of William T Piper Memorial Airport (41.1361${}^{\circ }$ N, 77.4223${}^{\circ }$ W). Two aircraft are simulated, each alternately climbs and descends at a vertical speed of 0.5 $\;m\;s^{-1}$ between a minimum and maximum altitude limit. The simulation begins on 2018-12-03 at 16:00 UTC, the environment was characterized by winds from the southwest with an initial low cloud deck that later cleared.

**Figure 5:**
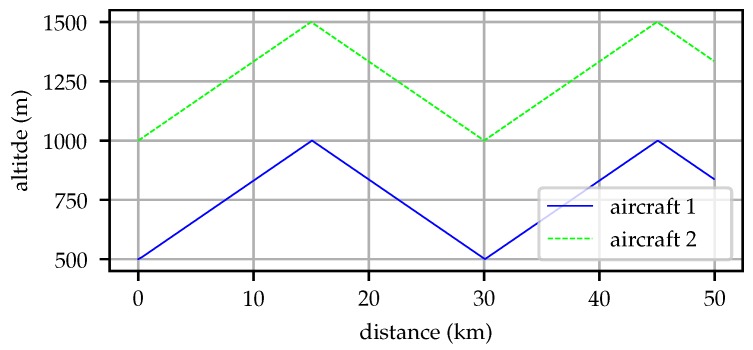
Flight paths taken by the two simulated aircraft for a simulation beginning at 16:00 UTC on 2018-12-03. One aircraft remains between 500 and 1000 m while the other stays between 1000 and 1500 m so neither can independently sample the region of interest.

The first aircraft has a minimum altitude of 500 m above sea level and maximum of 1000 m, the second aircraft has a minimum altitude of 1000 m and a maximum altitude of 1500 m. Neither aircraft reaches every point in the full region of interest (500–1500 m, mean sea level (MSL)) so they must communicate to achieve an understanding of the atmospheric structure. Figure 5 illustrates the longitudinal trajectory taken by the two aircraft.

Each aircraft builds a model of the profile of wind and solar fraction, at the simulation initialization each aircraft initializes its model to a zero mean state with an initial covariance equal to the climatological variance of the wind and solar fraction ($65\;m^{2}\;s^{-2}$ and 0.12 respectively, computed from the model grids discussed in Section 2.2). The aircraft take samples at 10 Hz and share their observations of the environment which are corrupted by sensor and sampling noise as described in Section 2.4 and Section 2.5. Each aircraft also updates its model of the environment using a HRRR forecast to provide a prediction of the change in atmospheric conditions in time and as the aircraft travels, time updates are performed every five minutes during the simulation.

### 4.1. Comparison between Aircraft

**Figure 6:**
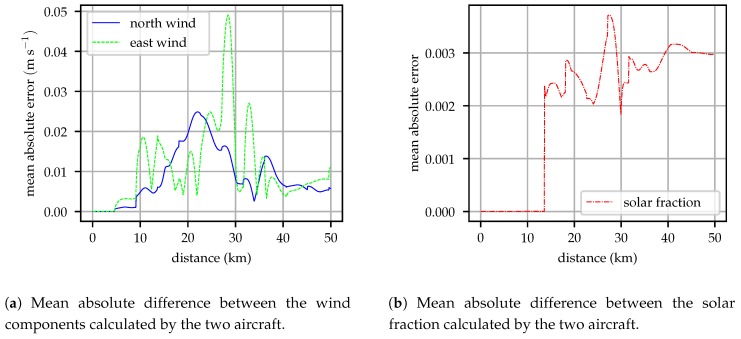
Mean absolute difference between the coefficients of the wind and solar models computed by the two aircraft as a function of the travel distance for a simulation beginning at 16:00 UTC on 2018-12-03. During time updates, slight differences in position and inertial velocity introduce small differences between the models maintained by each aircraft.

Under conditions of perfect communication, the aircraft should independently build identical maps of the environment. The observation model in the Kalman filter discussed in Section 2.6 cannot distinguish between an observation from the local aircraft or a remote measurement. Figure 6 illustrates the mean absolute error difference between model coefficients estimated by each aircraft for the wind vector components and solar fraction. Small differences between the two models are observed, this difference is introduced in the time update step of the Kalman filter. Each aircraft updates its state using Equation (5), computing the total derivative of the atmospheric state using a HRRR model grid provided before flight. Evaluating the total derivative requires the aircraft position and inertial velocity, small differences in aircraft position and speed introduce the differences observed in Figure 6.

During this flight, the mean wind vector was approximately 10 $\;m\;s^{-1}$, so the difference between models represents less than 1% of the wind magnitude. The solar fraction varied between 0.2 and 0.9 depending on altitude during this flight so the difference between the models constructed by each aircraft represents no more than 2% of the solar fraction. When the observed solar insolation is very small, the time update performs a random walk update rather than projecting the state forward from the forecast to avoid numerical precision issues. This is the case early in the flight when the aircraft are flying in clouds, once aircraft 2 reaches the illuminated region above the clouds its time update includes information from the forecast, causing the jump in error at 13 km.

### 4.2. Comparison to True Atmospheric State

In the simulation the true atmospheric state is available, permitting evaluation of the accuracy of the atmospheric modeling technique. During this flight, a significant change in cloud cover was observed. At the beginning of the flight a low-level cloud deck obstructed solar radiation at altitudes below approximately 1300 m. This cloud layer “burned off” during the flight and clear skies were later observed.

**Figure 7:**
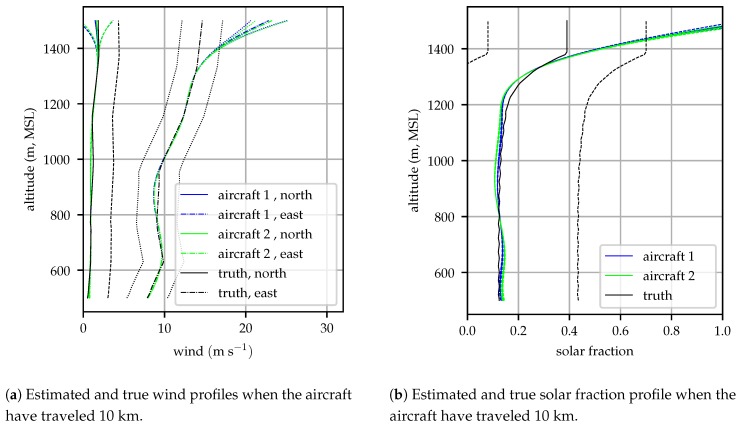
Estimated and true atmospheric profiles and 1σ bounds at 10 km into the flight for a simulation beginning at 16:00 UTC on 2018-12-03. Error bounds on the true profiles indicate the HRRR skill–the best that the atmospheric state can be known *a priori*.

Figure 7 shows the wind and solar fraction profiles and 1σ bounds estimated by both aircraft after flying 10 km. Error bounds are drawn for both the aircraft and truth profiles, the bounds on the true profiles represent the skill of the HRRR forecast model–the expected error in a forecast and limit to the performance of an *a priori* forecast [36,37]. At this time, the upper aircraft (“aircraft 2”) was at approximately 1250 m and had not yet sampled the highest altitudes so significant error can be observed around 1500 m, accompanied by relatively large model covariance. The lower aircraft (“aircraft 1”) was at 850 m, so there was a second unobserved region between 850 and 1000 m, but observations made by aircraft 2 above 1000 m allow the profile in this unobserved region to be inferred. In general, the estimated profiles track the true profiles well, with some error introduced because the spline cannot perfectly follow the profile which has discontinuous first derivative as it was interpolated from a discrete grid. There is no visually discernible difference between the profiles estimated by the two aircraft.

**Figure 8:**
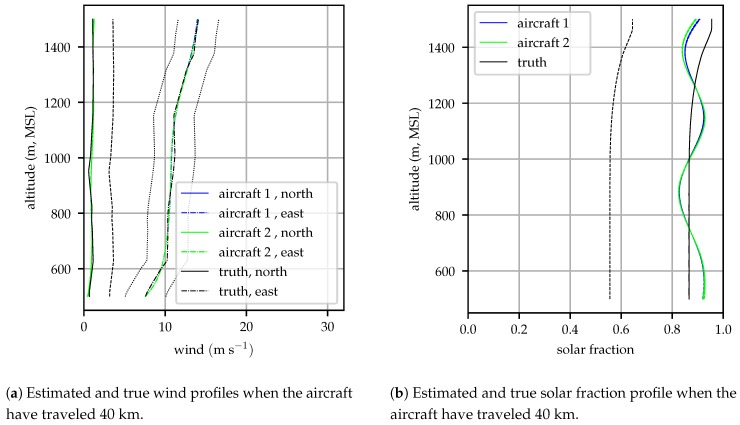
Estimated and true atmospheric profiles and 1σ bounds at 40 km into the flight for a simulation beginning at 16:00 UTC on 2018-12-03. Error bounds on the true profiles indicate the HRRR skill–the best that the atmospheric state can be known *a priori*.

Figure 8 shows the wind and solar fraction profiles estimated by the aircraft after traveling 40 km. By this time, the low cloud layer had dissipated, and nearly sunny conditions prevailed. The in situ modeling system continues to track the wind profile closely but shows greater error in the estimated solar profile; over much of the profile the true profile lies outside of the model’s 1σ bounds. This is because the process noise was chosen to reflect the average rate of change in the atmospheric conditions, whereas in this instance a rapid change occurs (clearing stratocumulus clouds). The estimated solar fraction at some altitudes reflect observations made by the aircraft while the sky was still partially clouded, introducing undulations into the estimated profile. Please note that even with these undulations the estimated solar profile is within 0.1 of the true value at all altitudes whereas the standard error for the HRRR model is 0.3 for the solar fraction [37].

Even with a significant shift in the atmospheric state during the course of a flight, the in situ modeling system is able to incorporate observations of the environment, maintaining a model which demonstrates less error than the forecast skill achieved by high-resolution weather forecasting systems, this can be seen in the figures by noting that the difference between the estimated and true profiles is always smaller than the error bounds drawn around the true profiles.

## 5. Flight Test

**Figure 9:**
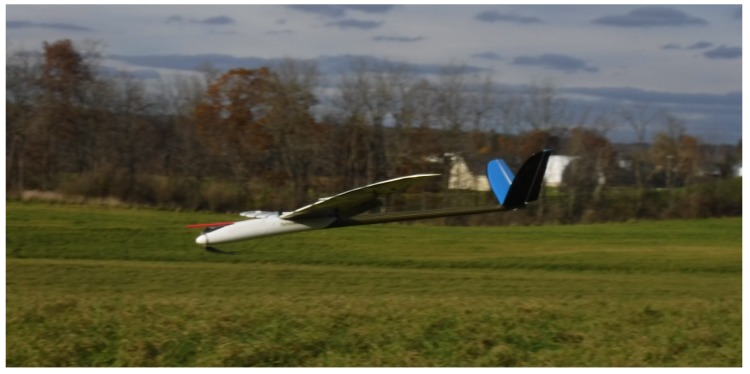
The Vulture, a small fixed-wing UAS based on a 2.5 m span model sailplane. It is equipped with an autopilot, onboard computer, and dual power systems permitting 30 min of flight time and more than 10 h of avionics run time. This allows multiple consecutive flights without disrupting the modeling system.

To test the environmental modeling and cost estimation system it was implemented on the Vulture, a small fixed-wing UAS depicted in Figure 9. The Vulture is equipped with a Pixhawk autopilot running the PX4 flight stack to provide low-level stabilization and control of the vehicle. It also carries an ODroid C1+ companion computer running the Robot Operating System which generates high level commands for the autopilot and runs the environmental modeling and cost estimation algorithm described in Section 2 and Section 3. The ODroid C1+ is a Linux/Android single-board computer with ARMv7 1.5GHz quad-core CPU and 1Gb RAM. A similar architecture is described in [38]. It has proven capable of real-time mapping, state estimation, planning, and control; further, all data collected is logged and stored to an eMMC flash storage.

Because the Vulture is not equipped with solar arrays, the environmental model used in flight tests only tracks the wind vector. Additionally, flight testing the insolation model would require flight in and above clouds, which is not currently possible under FAA Part 107 operations.

Both numerical weather model analyses and radiosondes launched during the test are used to validate the environmental model that is generated on board the aircraft.

**Figure 10:**
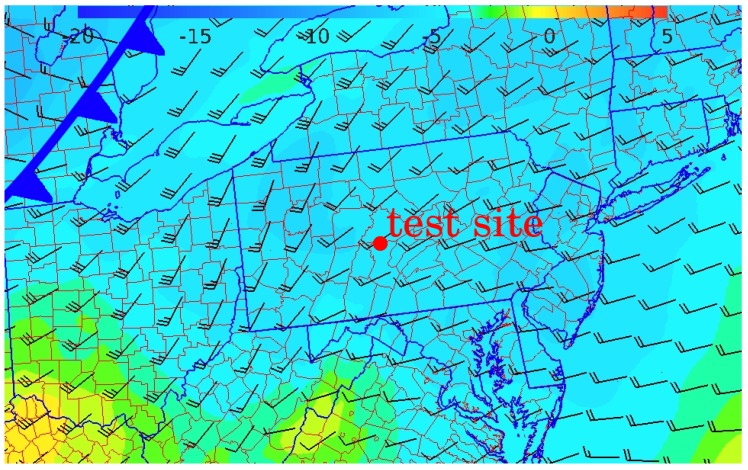
Wind and temperature at 925 mbar (approximately 750 m MSL) at 12:00 EST on December 6, 2018, approximately the midpoint of the test. The test site is located at the red dot, the approaching front can be seen as an abrupt wind shift along the eastern border of Michigan.

The test was conducted on 6 December 2018 in central Pennsylvania. The synoptic conditions featured a high-pressure system to the south-southwest and a weak cold front approaching from the west. Passage of the cold front was approximately 12 h after the completion of the flight test. Winds were from the southwest at approximately 7.5 $\;m\;s^{-1}$ as depicted in Figure 10.

### 5.1. Test Description

**Figure 11:**
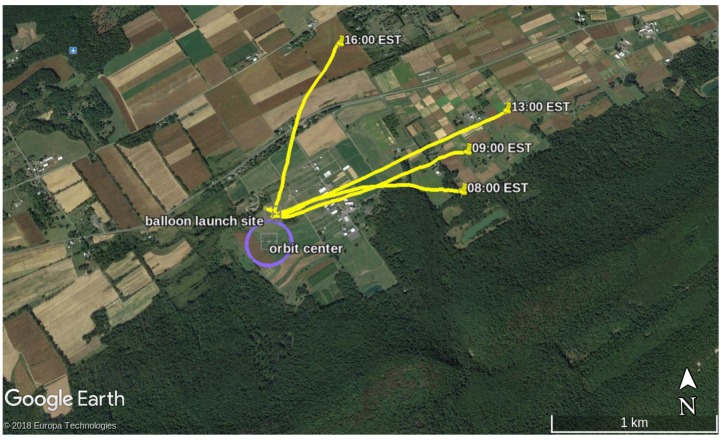
Aerial view of the test area showing the balloon trajectories and aircraft orbit location.

**Figure 12:**
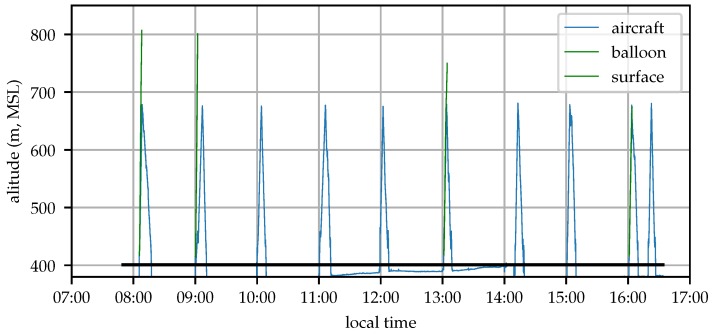
Time history of aircraft and balloon altitude (2018-12-06). The aircraft appears to be below ground between flights because its state estimator is incorporating airspeed measurements which are not informative when on the ground.

The wind profile was sampled by the aircraft once per hour beginning approximately one hour after sunrise and continuing until about half an hour before sunset. Each flight was conducted using identical pre-planned flight paths consisting of spiral climb and descent around a fixed GPS waypoint. Flights began with a constant-altitude orbit with radius 150 m at an altitude of 439 m MSL, roughly 30 m above ground level (AGL). The aircraft then climbed at 1.5 $\;m\;s^{-1}$ to an altitude of 674 m MSL (270 m AGL), then descended at approximately 0.75 $\;m\;s^{-1}$ (the aircraft’s descent rate with the motor shut down) until reaching the start altitude. Figure 11 shows the flight area and nominal aircraft orbit, Figure 12 illustrates the aircraft altitude during the test.

Several times throughout the day a Sparv Embedded S1 Windsonde was launched on a balloon from a site approximately 200 m north of the orbit point. A GPS sensor on the radiosonde provided drift information, which was used to determine wind speed, data was telemetered to the ground at 1 Hz. The radiosonde ascent was terminated by cutting the radiosonde free from the balloon after reaching 680 m, permitting the radiosonde to be retrieved and reused. Balloon profiles were taken concurrently with the aircraft profiles at 08:00, 09:00, 13:00, and 16:00 local time.

The wind profile modeling system was enabled throughout the entire test so that the model mean and covariance was propagated in between profiling flights. The time derivative of the wind profiles was obtained by numerically differentiating the 09:00 UTC (04:00 EST) HRRR forecast. When the aircraft’s airspeed was less than 10 $\;m\;s^{-1}$ measurement updates were disabled to prevent nonsensical wind measurements from being incorporated while the aircraft was on the ground.

### 5.2. Process and Measurement Noise

At each measurement step a “wind measurement” is synthesized by solving the wind triangle given inertial and air relative velocity [22]. The wind sensor noise is computed at each step from the wind measurement dynamics and the measurement noise of the sensors used in computing the “wind measurement” [22].

Averaged over 900 HRRR runs, the 1 h forecast error variance at the model grid point nearest the flight location is 0.95 $m^{2}\;s^{-2}$. The wind process noise is set so that the spline model state variance increases by this amount after one hour of time updates.

### 5.3. Results

**Figure 13:**
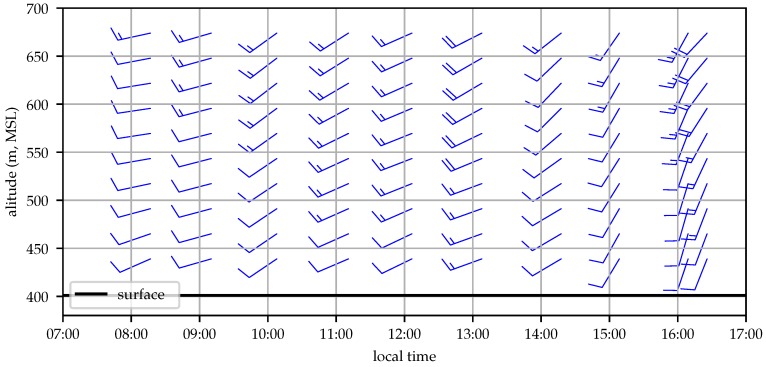
Time-altitude depiction of the profile of aircraft-estimated horizontal wind depicting its evolution throughout the day (2018-12-06).

Ten profiling flights were conducted by the aircraft, covering a time period of 8.5 h. The estimated wind profiles can be compared to those gathered by weather balloons and the analysis values from the HRRR. The wind profiling system runs in real time on board the aircraft and simultaneously stores all measurements (GPS time, position, and velocity; air relative speed; and aircraft attitude, acceleration, and angular rates) to a data log on the EMMC flash drive. After the flight tests a bug was found in the profiling system. That bug was fixed, and the results presented here were generated by running the stored flight data through the profiling system on the aircraft’s single-board computer. Figure 13 illustrates the evolution of the estimated wind profile throughout the day.

The wind profile initially shows increasing wind, likely a result of the deepening boundary layer drawing in momentum from the free troposphere above. Around 14:00 local time the wind magnitude decreases before the wind turns more southerly and increases in speed as the cold front approaches [39].

#### 5.3.1. Comparison with Radiosonde Observations

Comparisons between the radiosonde and aircraft measured winds can be used to determine that the aircraft is a reliable instrument for sampling the wind field [40,41]. The balloons were launched about 200 m from the orbit center point and drifted to the northeast, sometimes a kilometer or more before completing their profile. As the balloons ascended the radiosondes were observed swinging on their tethers, this motion sometimes interrupted GPS lock so the recorded data was quality controlled before analysis. Data points where the GPS lock or wind velocity was flagged as invalid were removed.

To simplify comparison to the aircraft measured profiles a basis spline with the same knot locations and order was fit to the radiosonde measurements. Detailed sensor specifications were not available for the radiosonde and the motion of the radiosonde introduces an additional error (i.e., the sensor package swinging on its tether beneath the balloon), making definition of the sensor variance difficult. The sensor noise was estimated from variance of the error between radiosonde wind samples and the mean wind profile estimated for each launch.

Because the balloon advects with the mean wind speed, it violates Taylor’s simplification for analyzing turbulent wind fields [24]. For the balloon, turbulent eddies do not appear as measurement noise, but as a bias on the entire profile. Reflecting this, the balloon wind variance is increased by $\frac{2}{3}tke$ to reflect the wind profile sample error. Please note that this use is different from using tke to represent the sample error in each wind measurement.

**Figure 14:**
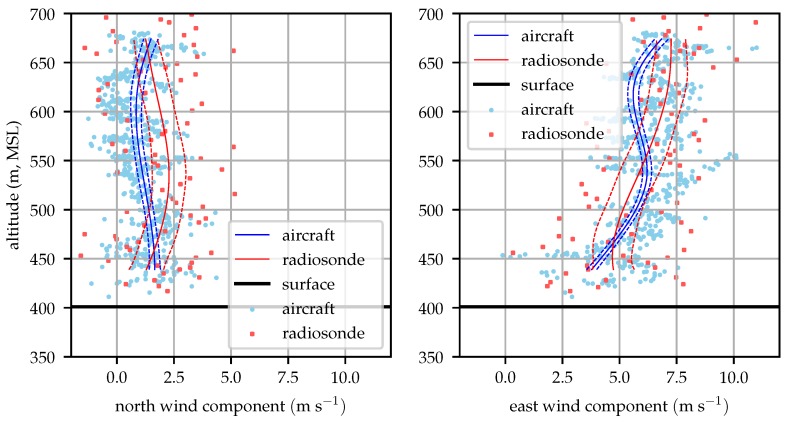
Aircraft and radiosonde profiles from 08:00 local time (2018-12-06). Solid lines represent the mean profile estimated by the aircraft and radiosonde while dashed lines represent the 1σ error bounds. The raw aircraft and radiosonde observations are depicted with scattered points.

**Figure 15:**
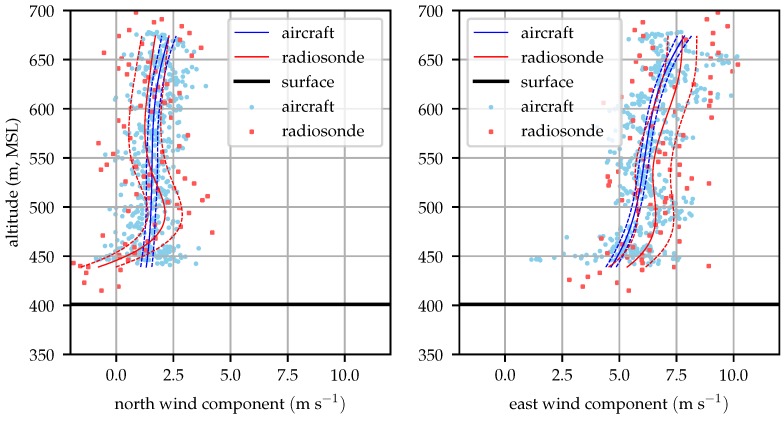
Aircraft and radiosonde profiles from 09:00 local time (2018-12-06).

**Figure 16:**
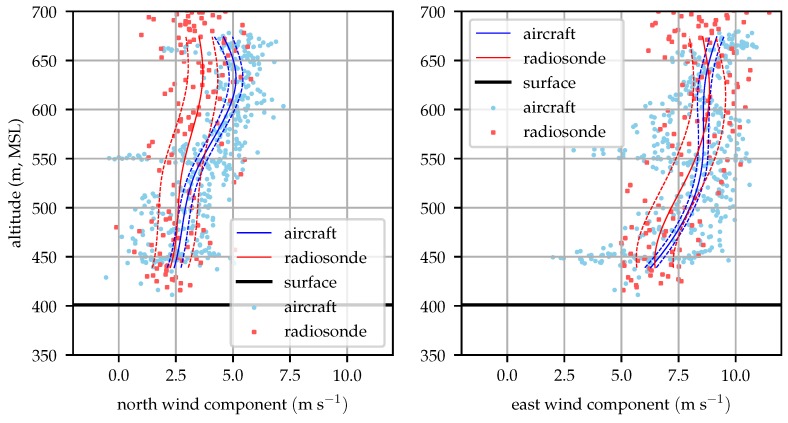
Aircraft and radiosonde profiles from 13:00 local time (2018-12-06).

**Figure 17:**
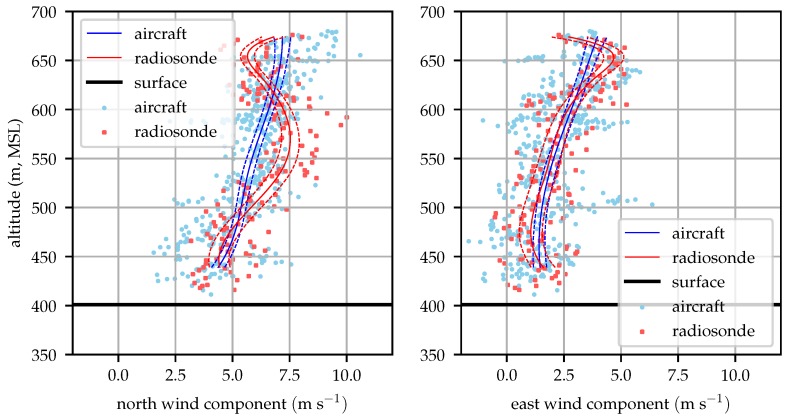
Aircraft and radiosonde profiles from 16:00 local time (2018-12-06).

Figure 14, Figure 15, Figure 16 and Figure 17 compare the aircraft filtered and radiosonde measured profiles at four times in the day. In each figure the mean profile and 1σ bounds are depicted for both the aircraft and radiosonde. Error bounds on the aircraft’s profile are determined by the Kalman filter. Please note that these bounds reflect the variance in the mean profile, not the wind observations. The observations are not expected to fall within the 1σ bounds 63% of the time, rather the mean profile will lie within the error bounds 63% of the time. The bounds are illustrated as an evaluation of the likelihood that the two techniques reflect observations of the same process and that the estimation system is producing a reasonable estimate for the wind profile variance.

Figure 14 shows that the mean profiles measured by the radiosonde and aircraft agree well, generally within their mutual 1σ error bounds. The raw radiosonde data are conspicuously sparse. As the balloon rises at approximately 3 $\;m\;s^{-1}$, the radiosondes provide data at 1 Hz so they only take about 100 samples before rising above the test area. Conditionally sampling for valid GPS lock reduces this data set by about 15%. The aircraft samples in contrast are taken at approximately 10 Hz and include the descending leg of the sampling mission.

Figure 15 compares radiosonde and aircraft measured profiles at 09:00 local time. Good agreement between the two is seen at altitudes above 450 m (80 m above ground).

Figure 16 shows good agreement between the radiosonde and aircraft profiles throughout most of the profile.

Figure 17 shows an unusual pattern in the radiosonde data, particularly in the north wind component. This perturbation is likely the result of the balloon advecting along with a particularly large eddy. Similar magnitude perturbations can be seen in the raw aircraft observations in Figure 16 and Figure 17. The aircraft can fly out of the eddy however, and the eddy is advected away from the flight area by the mean wind while the balloon drifts with these perturbations.

Nominally, this should be reflected by the magnitude of tke, but there is a source of turbulence that is not fully captured by the HRRR. A mountain ridge approximately 250 m tall is located about 1 km south and west of the test site, the ridge can be seen in Figure 11 running from southwest to northeast. As winds shifted around to the south, there would be a significant cross-mountain component resulting in shear-generated turbulent eddies shed off the mountain. The scale of this mountain system is of the same order as the grid resolution of the HRRR forecast model, so the effects of the mountains on the mean wind profile and tke is not fully resolved.

#### 5.3.2. Comparison with a Numerical Weather Model

**Figure 18:**
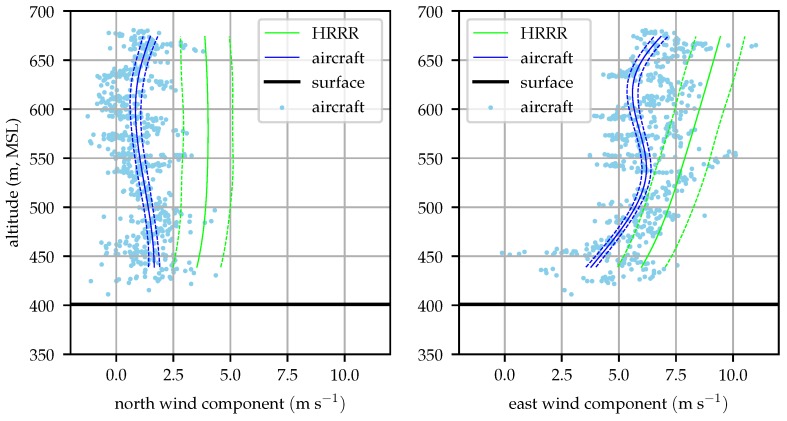
Aircraft measured and HRRR predicted wind profiles at 08:00 local time (2018-12-06).

**Figure 19:**
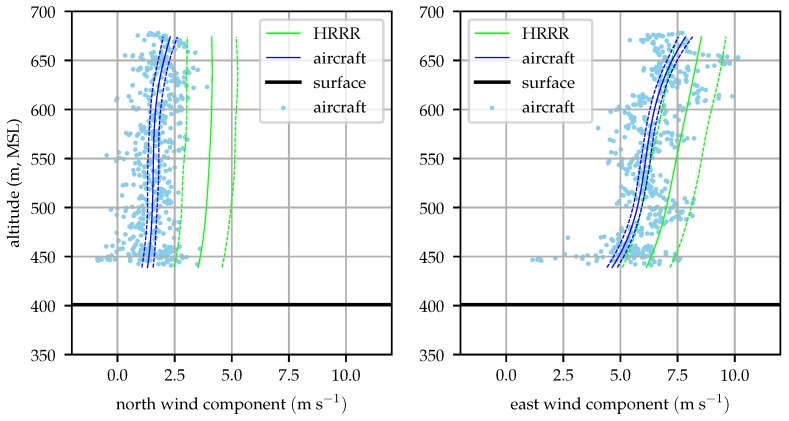
Aircraft measured and HRRR predicted wind profiles at 09:00 local time (2018-12-06).

**Figure 20:**
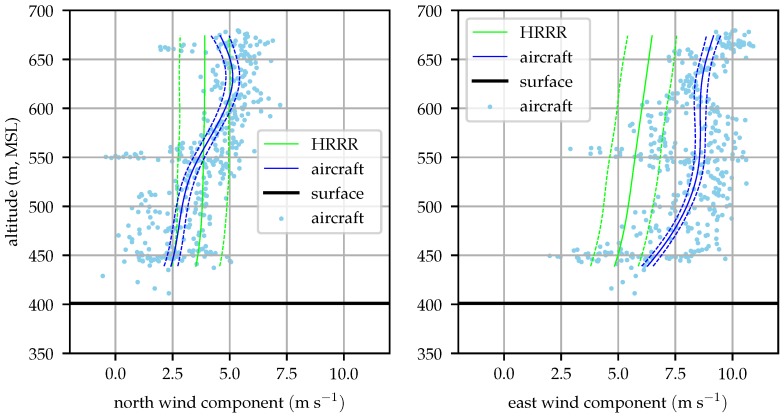
Aircraft measured and HRRR predicted wind profiles at 13:00 local time (2018-12-06).

**Figure 21:**
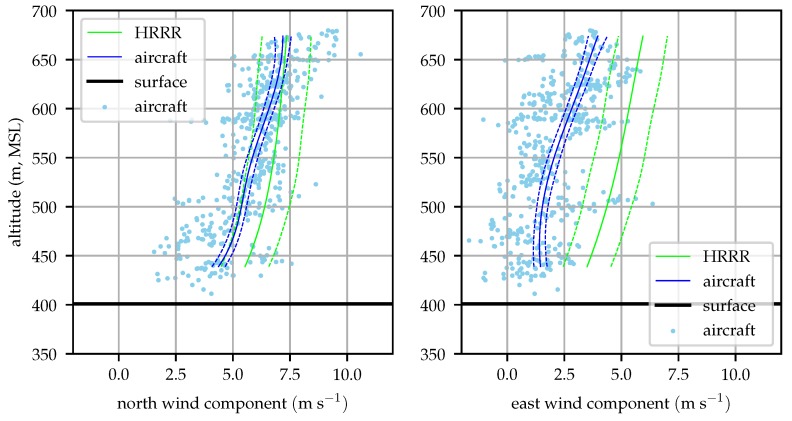
Aircraft measured and HRRR predicted wind profiles at 16:00 local time (2018-12-06).

The aircraft profiles were begun approximately on each hour to permit easy comparison with hourly weather model analyses. Figure 18, Figure 19, Figure 20 and Figure 21 compare the evolution of the aircraft and HRRR wind profiles at the hours nearest radiosonde observations. The 1σ bounds are depicted on both the aircraft and HRRR profiles.

It is worth noting here that the comparisons are made against the HRRR analyses obtained post-flight. The HRRR profiles depicted are not forecasts, but the best estimate of the atmospheric state that can be made by the fusing the model state with observations from meteorological sensors (balloons, weather stations, satellites, etc.) Using analysis data in flight would require considerable processing power on the ground be dedicated to providing very frequent analyses, and a constant high bandwidth link to the aircraft to update its data. This comparison then, represents the best-case for using offboard numerical weather prediction, less frequent updates from a forecast would likely perform worse.

The HRRR model error cannot be readily assessed from the either a single model output or a series of model runs. It is necessary to know the model’s analysis skill, the average error between the expected conditions and those actually observed. Benjamin et al. and Pichugina et al. indicate that the HRRR wind analysis skill is approximately 2.5 $\;m\;s^{-1}$ [36,42].

Figure 18 compares the aircraft measured profile after the 08:00 flight with the HRRR analysis at that time. The profiles show similar shapes but the HRRR profiles are 2–3 $\;m\;s^{-1}$ greater in both components. The undulation in the aircraft modeled east wind component observed by the aircraft between 500 and 600 m is likely the result of an eddy generated by either buoyantly rising air parcels or by the influence of surrounding terrain.

The effect of small eddies appear as random noise in the aircraft observed winds and are accounted for by increasing the measurement noise by the $\frac{2}{3}tke$. For this noise to be random and uncorrelated however, the aircraft must be traversing the wind field fast enough that the measurements are not correlated. The largest turbulent eddies in the boundary layer have a characteristic scale of roughly 1 km [14]. An aircraft which is circling in the same location is traversing the wind field on average at the wind speed. Given the wind magnitude during this test, measurements could be correlated for up to two minutes, so some of the eddies can appear as biases in the estimated wind field rather than noise, as seen in Figure 18. Aircraft traveling to a destination will traverse the wind field much faster, so sample to sample correlation is decreased and these perturbations will be eliminated.

Figure 19 clearly demonstrates the need to incorporate in situ observations to accurately determine the environmental state. The HRRR and aircraft measured profiles agree well in shape, but the HRRR over-predicts both wind components by approximately its analysis skill. At an altitude of 500 m, HRRR anticipated wind magnitude is about 2 $\;m\;s^{-1}$ greater than the measured profile, approximately 15% of the aircraft’s nominal cruise speed.

Figure 20 again shows the limitations of relying solely on numerical weather prediction models for understanding the aircraft’s environment. The east wind component shows the HRRR to be in error by 2–3 $\;m\;s^{-1}$, but this time *under predicting* the wind magnitude. This demonstrates that even if some validation data is available to develop a correction to the output of a numerical weather prediction system, it must be fairly available fairly frequently and at high resolution. In practice, it is simpler to estimate the atmospheric state onboard the aircraft.

Figure 21 shows the wind profile at 16:00 local, the second-to-last aircraft observed profile and last profile sampled in an hour. The wind is more southerly than anticipated by the HRRR, indicating that the prefrontal southerly winds are developing sooner than expected.

### 5.4. Travel Cost

While knowledge of the atmospheric structure may be useful in itself, the objective of this work is to determine the structure of mission performance cost. The transformations developed in Section 3 can be applied to the profiles to determine the cost profile.

**Figure 22:**
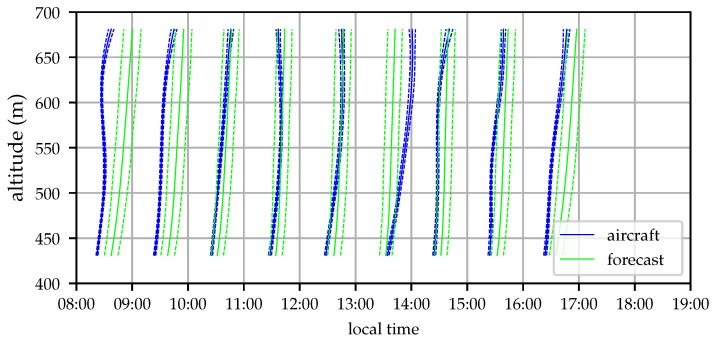
Evolution of the forecast and aircraft-estimated cost of traveling southwest at a groundspeed of 15 $\;m\;s^{-1}$ (2018-12-06). Cost profiles are scaled such that one hour represents 4 $\;m\;s^{-1}$ of specific power required to fly this mission. Systematic, slowly varying differences between the forecast and estimated cost profiles are seen.

Figure 22 illustrates the evolution of the cost profile for an example mission requiring the aircraft to fly southwest at a groundspeed of 15 $\;m\;s^{-1}$. The aircraft-estimated and forecast cost profile and covariance is shown once per hour. Cost profiles are plotted at each hour and scaled so that a profile reaching the next hour requires 4 $\;m\;s^{-1}$ of specific power output to fly this mission. In six of the ten cases the forecast cost profile differs from the aircraft-estimated cost profile by more than the forecast profile’s 1σ bounds. The difference between the forecast and estimated cost profiles sometimes exceeds 30% of the cost to accomplish the mission.

Evolution of the profiles illustrates that forecast error is systematic and slowly varying over the mission. Initially the forecast significantly over-predicts the cost to accomplish the mission (though it should be noted that it would under-predict the cost to travel in the opposite direction). Over the first five hours of the test this error slowly shrinks until at 12:00 local time the forecast cost is quite close to the aircraft-estimated cost. A transient change in the atmosphere at 13:00 EST (one that is observed by both the aircraft and radiosonde but not forecast) increases the cost during this hour and the forecast under-predicts the mission cost. Over the next few hours until the end of the test, the forecast cost begins growing and again over-predicts mission performance cost relative to the profile estimated by the aircraft.

**Figure 23:**
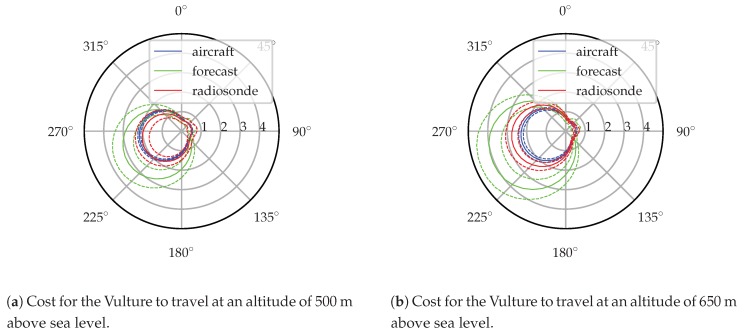
Cost for the Vulture to travel in any direction at an average groundspeed of 15 $\;m\;s^{-1}$. Cost is expressed in specific power and evaluated at 08:00 local time (2018-12-06).

To illustrate the ability of this system to change cost functions mid-flight Figure 23 shows the cost, expressed as specific power, and its 1σ bounds for the Vulture to travel in any direction at a groundspeed of 15 $\;m\;s^{-1}$ at 08:00 local time. Two altitudes are displayed, 500 and 650 m MSL. Every direction on the polar plot represents a different potential mission, the cost to accomplish any of these missions can quickly be determined by transforming the atmospheric profile into a mission cost performance profile.

Figure 23 also illustrates how in situ information can enable intelligent decision-making. Consider the earlier example mission (flying southwest at a groundspeed of 15 $\;m\;s^{-1}$). The forecast expects a significant increase in mission performance cost between 500 and 650 m altitude. The aircraft-estimated and balloon-observed profiles show considerably less difference in cost, and a lower overall cost to accomplish this mission. This information could enable an autonomous system or human operator to direct the aircraft to fly at a higher altitude and continue the mission longer, increasing terrain clearance and allowing the aircraft to cover a larger area if performing a mapping mission.

## 6. Conclusions

Basis splines provide a nonparametric way to model the atmospheric environment in a computationally efficient way. Since they are linear models they can easily be implemented in a Kalman filter, allowing simple and efficient updates to the model. This efficiency permits the atmospheric environment to be modeled using the limited computational resources available onboard a small UAS. The Kalman filter structure also permits distributed sensing systems if observations are available from other aircraft or remote sensing systems.

To accurately determine the covariance of the mean atmospheric structure, filter noise must include more than just the sensor characteristics. This additional error captures the sample variation from the mean state due to random fluctuations in the atmospheric state. The sample variance can be estimated from scaling laws or from the output of numerical weather prediction models.

Modeling the environment using basis splines permits the atmospheric state to be easily transformed into a cost for use in flight planning. The use of a Kalman filter to model the environment allows the environmental state covariance and thus cost covariance to be determined. The Kalman filter also permits fusing weather forecasts with in situ observations from the aircraft, a distributed flock of aircraft, or remote sensing systems. For linear transformations between atmospheric properties and cost, the cost profile and covariance is easily determined. Nonlinear transformations pose some additional difficulty, but the mean and covariance of the cost can be approximated using best-fit and nonlinear covariance transformation techniques.

In simulations the modeling system is demonstrated to accurately model the wind vector and solar insolation profiles using observations from multiple aircraft. Models constructed independently by two aircraft agree with only very small differences between them and the shared measurements enable each aircraft to model regions it cannot observe independently. The model tracks changes in the environment and estimates the atmospheric state with less error than high-resolution weather forecasting models achieve.

The wind profile filter was implemented on a small UAS and ten sampling missions conducted over the course of a day to track the evolution of the wind profile. The estimated wind profile is compared to numerical weather model analyses and to observations made with balloon-borne radiosondes. The aircraft-estimated and radiosonde-observed profiles generally agree up to their respective model covariances. The analysis profiles show greater deviation, frequently exceeding even the analyses’ greater profile variance.

Propagating the wind model obtained by the aircraft through a cost function allows the aircraft to determine the mean cost to travel in a desired direction and the confidence in that cost. Throughout the day, systematic differences are observed between the forecast and estimated cost to accomplish an example mission. The in situ estimate of the environment improves the aircraft’s situational awareness and can enable a human operator or automated scheduler to weigh the cost of an action. In one example from the flight test, the onboard modeling system shows that the cost to accomplish an example mission changes little with altitude while the forecast expects the cost to be greater overall with a significant penalty for flying higher. In this case, in situ information provides the operator with better information about the mission performance cost and gives greater operational flexibility by showing that the mission can be performed for similar cost at several altitudes.

Fusing *a priori* information available from numerical weather prediction models with observations made in situ by an aircraft provides a method to improve the quality of atmospheric state information available to the aircraft without requiring significant ground computation resources or constant communications links to the aircraft. The proposed model structure allows distributed sensing systems while providing a self-contained ability for individual aircraft to determine the profile of their mission performance cost.
